# Patient harm associated with serial phlebotomy and blood waste in the intensive care unit: A retrospective cohort study

**DOI:** 10.1371/journal.pone.0243782

**Published:** 2021-01-13

**Authors:** Thomas Bodley, Maverick Chan, Olga Levi, Lauren Clarfield, Drake Yip, Orla Smith, Jan O. Friedrich, Lisa K. Hicks

**Affiliations:** 1 Interdepartmental Division of Critical Care, University of Toronto, Toronto, Ontario, Canada; 2 Department of Medicine, University of Toronto, Toronto, Ontario, Canada; 3 Li Ka Shing Knowledge Institute, St. Michael’s Hospital, Toronto, Ontario, Canada; 4 Division of Laboratory Medicine, St. Michael’s Hospital, Toronto, Ontario, Canada; 5 Division of Hematology/Oncology, St. Michael’s Hospital, Toronto, Ontario, Canada; University Magna Graecia of Catanzaro, ITALY

## Abstract

**Background:**

Intensive care unit (ICU) patients are at high risk of anemia, and phlebotomy is a potentially modifiable source of blood loss. Our objective was to quantify daily phlebotomy volume for ICU patients, including blood discarded as waste during vascular access, and evaluate the impact of phlebotomy volume on patient outcomes.

**Methods:**

This was a retrospective observational cohort study between September 2014 and August 2015 at a tertiary care academic medical-surgical ICU. A prospective audit of phlebotomy practices in March 2018 was used to estimate blood waste during vascular access. Multivariable logistic regression was used to evaluate phlebotomy volume as a predictor of ICU nadir hemoglobin < 80 g/L, and red blood cell transfusion.

**Results:**

There were 428 index ICU admissions, median age 64.4 yr, 41% female. Forty-four patients (10%) with major bleeding events were excluded. Mean bedside waste per blood draw (144 draws) was: 3.9 mL from arterial lines, 5.5 mL central venous lines, and 6.3 mL from peripherally inserted central catheters. Mean phlebotomy volume per patient day was 48.1 ± 22.2 mL; 33.1 ± 15.0 mL received by the lab and 15.0 ± 8.1 mL discarded as bedside waste. Multivariable regression, including age, sex, admission hemoglobin, sequential organ failure assessment score, and ICU length of stay, showed total daily phlebotomy volume was predictive of hemoglobin <80 g/L (p = 0.002), red blood cell transfusion (p<0.001), and inpatient mortality (p = 0.002). For every 5 mL increase in average daily phlebotomy the odds ratio for nadir hemoglobin <80 g/L was 1.18 (95% CI 1.07–1.31) and for red blood cell transfusion was 1.17 (95% CI 1.07–1.28).

**Conclusion:**

A substantial portion of daily ICU phlebotomy is waste discarded during vascular access. Average ICU phlebotomy volume is independently associated with ICU acquired anemia and red blood cell transfusion which supports the need for phlebotomy stewardship programs.

## Introduction

In the intensive care unit (ICU) blood testing guides diagnosis, monitoring, and titration of invasive therapies. However, previous reports suggest that a significant proportion of ICU blood tests are likely reflexive and unnecessary [1, 2]. Repetitive phlebotomy can increase complications from venipuncture [3], the risk of hospital acquired anemia [4, 5], the need for red cell transfusion [6], and may prolong hospital length of stay [5]. Furthermore, misleading results of inappropriate or unnecessary investigations can lead to a cascade of diagnostic tests and interventions [7, 8], which can contribute to diagnostic error and adverse events [9]. Two studies from inpatient populations show that hospital acquired anemia is a predictor of inpatient mortality [4, 10], suggesting clinicians may inadvertently cause harm through phlebotomy if it contributes to low hemoglobin.

The prevalence of anemia and harms from serial phlebotomy among critically ill patients were described over a decade ago [6, 11], yet only recently have international campaigns to reduce over testing gained traction [12, 13]. Interventions with the potential to curtail in-hospital phlebotomy include: provider education and policy interventions [14], audit and feedback [15], computerized decision support and order set changes [16], and smaller phlebotomy tubes or other blood conservation devices [17–19]. Coordinated multidisciplinary and multimodal quality improvement initiatives are effective [18, 20–23], though these interventions can be challenging to sustain. Furthermore, interventions to reduce phlebotomy are generally not linked to patient specific outcomes, fueling the controversy as to whether there is a modifiable link between phlebotomy and harm among hospitalized patients [24–26]. Better understanding the drivers of ICU phlebotomy, the potential for patient harm, and developing realistic targets for patient specific outcomes is important for planning, implementing, and evaluating stewardship interventions.

In this study, we characterize phlebotomy practices and patient outcomes in a tertiary, academic medical-surgical ICU. Our objectives were to describe phlebotomy practices, including waste discarded at the bedside during vascular access, and explore the relationship between phlebotomy volume and patient outcomes. We hypothesized that higher average daily phlebotomy during an ICU admission would be an independent risk factor for acquired anemia and the need for red cell transfusion.

## Methods

### Design, setting, and participants

This was a retrospective, observational cohort study of phlebotomy practices in a 24-bed medical-surgical ICU at a tertiary care academic hospital in Toronto, Canada. Index ICU admissions between September 1, 2014 and August 31, 2015 with a minimum ICU length of stay of 72 hours were included. Admissions shorter than 72 hours were excluded in the study protocol since the exposure time to ICU associated phlebotomy was short. Patients with an ICU length of stay extending beyond August 31, 2015, were excluded due to incomplete lab data. Major bleeding events were defined as a hemoglobin drop of 30 g/L within a 24-hour period, and patients with major bleeding events were excluded from multivariable outcomes analysis. The retrospective study was combined with a prospective audit in March, 2018, of bedside phlebotomy practices estimating blood waste during vascular access in the same ICU. Phlebotomy practices during the prospective audit were assumed to be similar to the study period since no changes in blood draw techniques were introduced in the interim.

### Data sources

Hospital administrative data was used to extract demographics, hospital and ICU length of stay, and discharge disposition including death. Administrative data from the laboratory information system provided the number and type of blood samples for each patient including the requested tests, test results, and the number and timing of red blood cell transfusions. Lab data and select chart review was used to calculate the day 1 Sequential Organ Failure Assessment (SOFA) score [27]. For patients who were intubated on admission the Glasgow Coma Scale was assumed to be 15/15 for SOFA score estimation [28].

### Outcomes and definitions

Primary outcomes of interest were 1) nadir hemoglobin less than 80 g/L during ICU admission, and 2) need for a red blood cell transfusion. A secondary outcome was in-hospital mortality. Independent variables used in logistic regression included: age, sex, ICU day 1 SOFA score, admission hemoglobin, ICU length of stay, and average daily phlebotomy volume.

Average daily phlebotomy volume included blood sent to the lab for diagnostic testing and blood discarded at the bedside during vascular access. The average daily volume of blood sent for diagnostic testing was determined on a per-patient basis by summing the total volume of blood sent for diagnostic testing during the ICU stay, divided by ICU length of stay in days. All lab samples were included in the analysis including venous, arterial, and blood culture samples. Manufacturer recommended tube volumes from the study period (September 2014 to August 2015) were used to convert number of tubes collected to blood volume collected in mL (tube volumes are available in S1 File). Average daily waste was calculated from the total number of unique blood draws during ICU admission, multiplied by the average waste per vascular access event, and divided by the ICU length of stay. Waste per vascular access event was determined using a prospective audit of ICU phlebotomy practices in March 2018. Average waste per draw was calculated using the proportion of blood draws from arterial, central venous, peripherally inserted central catheters (PICCs), and the average waste for each respective vascular device. Blood cultures were not included in the waste estimates as there is no discard volume required for these samples.

### Statistical analysis

Descriptive statistics were used to summarize baseline cohort characteristics. Continuous variables were summarized as mean (± standard deviation) or median (max/min value) for normally and non-normally distributed variables respectively. Characteristics of patients who received a blood transfusion were compared to those without transfusion using Student-t tests for continuous variables and Chi-squared tests for categorical variables where appropriate.

Multivariable logistic regression was used to study the association between average daily phlebotomy volume during ICU admission, nadir hemoglobin less than 80 g/L, and the need for red blood cell transfusion. Variables in the model included age, sex, ICU day 1 SOFA score, admission hemoglobin, and ICU length of stay. Results are reported as Odds Ratios (OR) with 95% confidence intervals (CI) with two-sided p values < 0.05 judged as statistically significant. A secondary analysis was conducted using in-hospital mortality as the dependent variable. Statistical analyses were conducted using Microsoft Excel 2019 (Microsoft Corp, Redmond, WA) and SAS version 9.4 (SAS Institute, Cary, NC).

### Sensitivity analysis

The multivariable logistic regression for primary and secondary outcomes was repeated using average daily phlebotomy volume excluding the estimate of waste during vascular access. Sensitivity of the definition of new ICU acquired anemia was also evaluated; regression models were repeated using nadir hemoglobin cut-off values of 90 g/L, 85 g/L, and 75 g/L. All sensitivity analysis results are reported in S2 File.

### Subgroup analysis

To facilitate direct comparison to prior work at our institution [6], patients with a ICU length of stay of 30 or more days were analyzed for average daily phlebotomy volume after ICU day 30 (with and without waste). This data is reported in the Results section.

### Research ethics statement

All data was anonymized prior to data manipulation and analysis. For chart review components, an independent master linking log was maintained separate from the primary database. Approval to waive informed consent was obtained and the study was approved through the institutional review board at St. Michael’s Hospital, Toronto, Ontario (REB #18–050).

## Results

Between September 1, 2014 and August 31, 2015, there were 963 index admissions to the medical-surgical ICU of which 428 patients had an ICU length of stay of 72 hours or more (5213 patient days). Table 1 summarizes baseline cohort characteristics and phlebotomy volumes. Average total daily phlebotomy volume was 48.1 ± 22.2 mL/day, of which 33.1 ± 14.2 mL/day was sent for laboratory testing and 15.0 ± 8.1 mL/day was discarded at the bedside during vascular access. The average number of blood draws was 3.5 ± 1.9 per patient-day. A total of 34% (1784/5213) of all patient days had at least five separate blood draws and 59% (3063/5213) had 3 or more. 9% (477/5213) of patient days had five arterial blood gas (ABG) samples and 26% (1346/5213) had 3 or more ABG samples. Cohort characteristics and markers of lab test utilization are summarized in Table 1.

**Table 1:** Cohort characteristics for ICU admissions from Sept 1, 2014 to August 31, 2015.

	All ICU Admissions (N = 963)	Included ICU Admissions (length of stay ≥ 72 hr) (N = 428)
Female, (%)	401 (42%)	174 (41%)
Median age, yr (max-min)	63.9 (97.6–17.3)	64.4 (97.6–18.5)
Inpatient mortality, (%)	158 (16%)	95 (22%)
ICU length of stay, days (SD)	6.6 (± 11.6)	12.2 (± 15.9)
Hospital length of stay, days (SD)	20.1 (± 46.1)	29.0 (± 49.1)
Required red cell transfusion, (%)	265 (28%)	176 (41%)
Average daily phlebotomy sent for testing, mL/day (SD)	30.4 (± 20.3)	33.1 (± 14.2)
Average daily blood draws, draws/d (SD)	3.1 (± 2.5)	3.5 (± 1.9)
Average daily blood waste from vascular access, mL/day (SD)	13.5 (± 10.8)	15.0 (± 8.1)
Total phlebotomy (testing + waste), mL/day (SD)	43.9 (± 31.1)	48.1 (± 22.2)
Patients with 5 or more discrete blood draws in one day, (%)	-	373 (87%)
Patients with 5 or more ABGs in one day, (%)	-	168 (39%)
Major bleeding events*^,^^†^, (%)	-	44 (10%)
Invasive mech. ventilation on ICU admission^†^, (%)	-	217 (51%)
Non-invasive mech. ventilation on ICU admission^†^, (%)	-	30 (7%)
Vasopressors on ICU admission, (%)	-	146 (34%)
Average day 1 SOFA score^†^^,^^‡^, (SD)	-	6.0 (± 3.2)

SD = standard deviation, ICU = intensive care unit ABG = arterial blood gas, SOFA = sequential organ failure assessment.

*Major bleeding definition: a drop in hemoglobin of > 30 g/L in a 24 hour period.

^†^Major bleeding events and SOFA scores were only extracted for patients with ICU length of stay ≥ 72 hours.

^‡^For invasively mechanically ventilated patients the Glasgow coma scale component of the day 1 SOFA score was assumed to be zero.

Table 2 compares patients who did and did not receive a red cell transfusion. Patients receiving a red cell transfusion (176/428, 41%) had higher average total daily phlebotomy volumes compared to those without transfusion (57.5 vs 41.6 mL, p<0.001), a longer ICU length of stay (15.6 vs 9.8 days, p<0.001), and were more likely to die in hospital (31% vs 16%, p<0.001).

**Table 2:** Comparison of patients with and without a red blood cell transfusion during their ICU stay.

	Red Blood Cell Transfusion (N = 176)	No Red Blood Cell Transfusion (N = 252)	p-value
Female, %	66 (38%)	108 (43%)	0.27
Median age, yr (max-min)	64.9 (97.6–24.7)	64.2 (94.7–18.5)	0.14
Inpatient mortality, %	54 (31%)	41 (16%)	<0.001
ICU length of stay, days	15.6 (± 15.2)	9.8 (± 16.0)	<0.001
Hospital length of stay, days	37.9 (± 62.5)	22.7 (± 35.7)	0.004
Average daily phlebotomy for testing, mL/day	39.6 (± 16.0)	28.6 (± 10.7)	<0.001
Average daily blood draws, draws/d	4.1 (± 2.1)	3.0 (± 1.5)	<0.001
Average daily blood waste with vascular access, mL/day	17.9 (± 9.1)	13.0 (± 6.5)	<0.001
Total phlebotomy (testing + waste), mL/day	57.5 (± 25.1)	41.6 (± 17.2)	<0.001
Major bleeding events*, %	32 (18%)	12 (5%)	<0.001
Invasive mech. ventilation on ICU admission, %	102 (58%)	59 (23%)	0.01
Non-invasive mech. ventilation on ICU admission, %	6 (3%)	24 (10%)	0.02
Vasopressors on ICU admission, (%)	87 (49%)	59 (23%)	<0.001
Average day 1 SOFA score^†^	7.4 (± 3.4)	5.0 (± 2.7)	<0.001

SD = standard deviation, ICU = intensive care unit ABG = arterial blood gas, SOFA = sequential organ failure assessment.

*Major bleeding definition: a drop in hemoglobin of > 30 g/L in a 24 hour period.

^†^For invasively mechanically ventilated patients the Glasgow coma scale component of the day 1 SOFA score was assumed to be zero.

During the March 2018 prospective bedside audit of phlebotomy practices, 132 blood draws were observed: 109 (76%) from arterial lines, 7 (5%) from central venous catheters, and 16 (11%) from PICCs. The average volume discarded at the bedside during vascular access varied by vascular device: 3.9 mL for arterial lines, 5.5 mL for central venous catheters, and 6.3 mL for PICC lines. The proportional utilization of different vascular access points and average waste per line was used to estimate blood waste per draw in the retrospective cohort. Full results of the prospective audit are available in S3 File.

**Fig 1:**
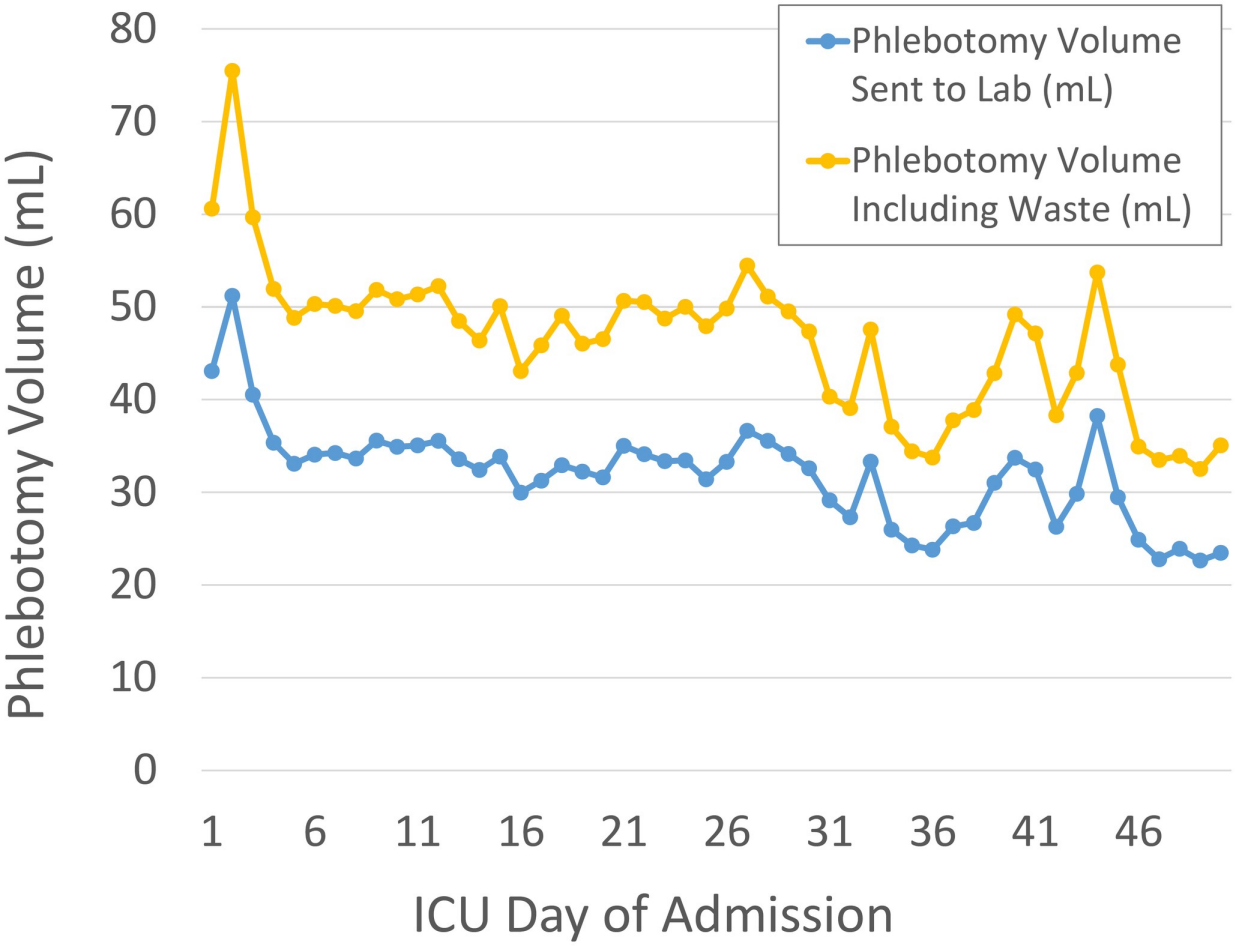
Phlebotomy volume by date from ICU admission with and without the estimates for waste during vascular access. N = 428.

**Fig 2:**
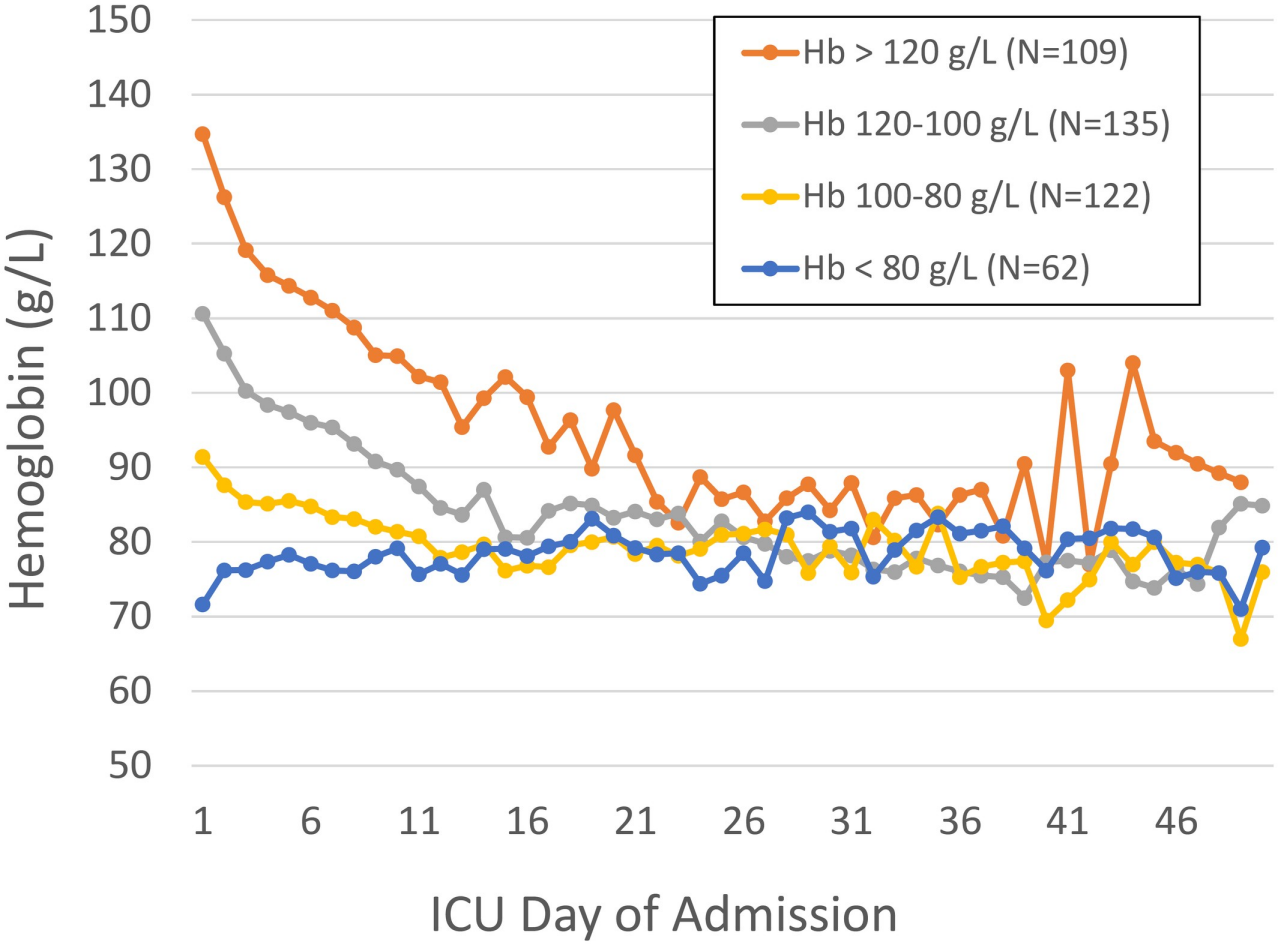
Hemoglobin trend by ICU day stratified by admission hemoglobin level. N = 428.

Fig 1 shows daily phlebotomy volume by day of ICU admission with and without waste. Fig 2 illustrates daily hemoglobin values for the cohort stratified by admission hemoglobin level.

Results of the multivariable logistical regression for primary outcomes of nadir Hb < 80 g/L, the need for red cell transfusion, and secondary outcome in-hospital mortality are provided in Table 3. For every 5 mL increase in average daily phlebotomy the odds ratio for nadir hemoglobin < 80 g/L was 1.18 (95% CI 1.07–1.31) and for red cell transfusion 1.17 (95% CI 1.07–1.28). Sensitivity analysis are provided in S2 File. ICU phlebotomy remained a statistically significant predictor of ICU acquired anemia using Hb < 75 g/L, < 85 g/L, and < 90 g/L as definitions in multivariable logistic regression.

**Table 3:** Multivariable logistic regression for continuous and parametric predictors of 1) nadir hemoglobin < 80 g/L, 2) red cell transfusion in the ICU, and 3) hospital mortality. N = 384 after exclusion of major bleeding events*.

	1) Nadir Hb < 80 g/L		2) Red Blood Cell Transfusion		3) Hospital Mortality	
	Odds Ratio Estimates (95% CI)**	P Value	Odds Ratio Estimates (95% CI)**	P Value	Odds Ratio Estimates (95% CI)**	P Value
Daily phlebotomy volume, per mL	1.03 (1.01–1.06)	0.002	1.03 (1.01–1.05)	<0.001	1.02 (1.01–1.04)	0.002
Age, yr	1.01 (0.99–1.04)	0.19	1.02 (1.00–1.04)	0.04	1.03 (1.01–1.05)	0.005
Sex (male)	0.71 (0.36–1.42)	0.33	1.05 (0.59–1.86)	0.87	1.05 (0.59–1.85)	0.87
ICU admission hemoglobin, g/L	0.91 (0.88–0.93)	<0.001	0.93 (0.92–0.95)	<0.001	1.01 (0.997–1.02)	0.13
ICU admission SOFA score^†^	1.17 (1.03–1.32)	0.01	1.16 (1.05–1.27)	0.003	1.10 (1.01–1.20)	0.04
ICU length of stay, d	1.21 (1.13–1.29)	<0.001	1.03 (1.01–1.06)	0.002	1.02 (1.00–1.03)	0.01

CI = Confidence Interval, ICU = intensive care unit, SOFA = sequential organ failure assessment.

*Major bleeding definition: a drop in hemoglobin of > 30 g/L in a 24 hour period.

^†^For invasively mechanically ventilated patients the Glasgow coma scale component of the day 1 SOFA score was assumed to be zero.

Subgroup analysis of patients with an ICU length of stay of 30 days or more (N = 35, median length of stay 44 days) demonstrated that the average ICU associated phlebotomy volume between day 30 and day 50 was 40.3 ± 19.3 mL (28.5 mL sent to the lab for testing and 11.8 mL bedside waste).

## Discussion

Laboratory testing is essential for the diagnosis, monitoring, and titration of invasive therapies in critical care. Our study observed that current ICU laboratory diagnostic phlebotomy practices involve collecting high volumes of blood per patient day (Table 1), especially when blood discarded as waste during vascular access is included. We also found a high frequency of discrete blood draw events, including over one third of all patient days having 5 or more blood draws per day. Multivariable logistic regression supports the hypothesis that phlebotomy contributes to ICU associated anemia and red cell transfusion.

Our study provides a contemporary assessment of phlebotomy practices in an academic tertiary care ICU in Canada. Average daily phlebotomy volume for laboratory testing was 33.1 mL/day, with blood discarded during vascular access contributing a further 15.0 mL/day. Our findings are similar to studies published nearly two decades ago; for example, in Europe across 145 sites in 2002 a prospective 24 hour audit found that average daily phlebotomy volume was 41.1 mL/day from a mean number of blood draws of 4.6 [11]. That such little apparent progress in reducing phlebotomy volume has been made in the intervening years is concerning.

Another Canadian study published in 2019 affords a more recent contrast [29]. Quinn and colleagues reported a mean ICU daily phlebotomy volume of 27.2 +/- 20.0 mL/day, about 18% lower than our estimate of blood volume used for lab testing. While the difference may be explained by variability between cohorts, study methodology, or local practice, the more notable difference is the importance of factoring in waste estimates for ICU patients. Including bedside waste increases our total daily phlebotomy to 48.1 mL/day, making our estimate of true phlebotomy volume nearly double that of Quinn and colleagues. The frequency of blood draws determines waste and is an important consideration in addition to testing volumes.

Blood conservation devices to eliminate vascular access waste have been proposed [30], with demonstrated efficacy including reduction of ICU acquired anemia [31]. However, such devices have not gained traction, partially owing to concerns over bacterial contamination [32], and the cost/complexity of implementation [33]. A recent systematic review suggests conservation devices may represent an under-utilized tool [19]. A more common approach to reducing phlebotomy is to decrease the number and frequency of tests [18, 20]. Our study suggests there is room for improvement in this regard; 34% of all patient days in our cohort had at least five separate blood draws, and 59% had 3 or more. While some patients require frequent blood monitoring, the frequency of testing seems high in our sample, and likely represents an opportunity for improvement such as through batching lab tests to reduce blood waste. Recent campaigns to reduce unnecessary investigations have been met with enthusiasm [12, 13]. However there is a growing recognition that raising awareness alone is not sufficient [34], and must be complemented by multifaceted concerted efforts to curtail phlebotomy [18, 20, 35]. Methods include batching lab samples, offering “add-on” test orders to prevent blood draws when additional tests are needed, and encouraging healthcare teams to assess the frequency of blood testing on daily rounds [20].

While curtailing unnecessary investigation is important, there may also be opportunities to reduce the volume of blood collected from ICU patients by reducing the volume of blood in collection tubes [17, 36]. Preliminary retrospective and prospective cohort studies suggest that this approach may be effective [37]. This approach is also supported in a recent systematic review of laboratory practices to reduce phlebotomy [19]. Randomized control trials of standard versus smaller vacutainer blood collection tubes in the ICU will be an important contribution to the literature when reported [38, 39]. Importantly, efforts to reduce tube sizes will not address the issue of blood wastage, and benefits from this strategy would be additive to any reduction in testing frequency. Thus, the potential to reduce tube size should be viewed as an adjunct to, not a replacement for, other stewardship initiatives aimed at decreasing the frequency of tests.

Our study suggests that phlebotomy volume is an independent predictor of ICU acquired anemia (Hb < 80 g/L) and the need for red blood cell transfusion. Similar findings have been reported in ICU and other acute cares settings [5, 6, 31], yet phlebotomy induced or iatrogenic anemia remains controversial [24–26]. Counter arguments include correlation rather than causation (the sickest patients require the most frequent laboratory testing) [24], and that other mechanisms drive anemia including impaired erythropoiesis in critically ill patients, and non-diagnostic phlebotomy related blood loss [40, 41]. Our study supports phlebotomy as a direct and substantial contributor to patient harm through anemia and increased transfusion requirements. In our multivariable logistic regression, increasing average daily phlebotomy by 5 mL of blood was a stronger predictor of ICU acquired anemia and the need for red cell transfusion than a 1-point increase in day 1 SOFA score. These findings were consistent with or without our estimate of bedside blood waste during vascular access. Results were also consistent regardless of the nadir hemoglobin threshold used to define ICU acquired anemia (from 70 to 90 g/L).

Our study has several limitations. First, our study is single center and may not be generalizable to other units—particularly to those with a very different population of patients such as a trauma, cardiovascular, or pediatric ICUs. However, the congruency of our findings with other tertiary care ICUs [29], supports the idea that similar phlebotomy practices are present elsewhere. Secondly, as a retrospective cohort our study may be confounded by unmeasured factors, for example it is possible that severity of illness was not adequately captured by the day 1 SOFA score. Our analysis also suggests that increased phlebotomy is independently associated with increased mortality. Although this is similar to findings of other large multi-centre observational studies of anemia in the ICU [11, 40], it may represent residual confounding. Thirdly, while our study includes patients with long ICU length of stay (eg. over 30 days) this population is limited due to our sampling method and findings related to long-stay patients should be interpreted with caution. Not-withstanding the above, the correlation between phlebotomy and patient harm warrants attention, further investigation, and ongoing consideration of phlebotomy stewardship. As monitoring ICU performance becomes increasingly common [42], our work supports phlebotomy volume as a potential quality metric to benchmark across institutions.

## Conclusions

In conclusion, higher average daily phlebotomy volume is associated with ICU acquired anemia and the need for red blood cell transfusion in a multivariable model accounting for demographics, major bleeding events, and severity of illness as estimated by day 1 SOFA score. The association between higher average daily phlebotomy volume and ICU mortality warrants further investigation. Our findings support the need for ongoing phlebotomy stewardship interventions in the ICU. We suggest ICU acquired anemia and the need for red blood cell transfusion are appropriate patient outcome measures to evaluate stewardship interventions.

## Supporting information

S1 File Manufacturer tube volumes. (DOCX)

S2 File Sensitivity analysis. (DOCX)

S3 File Waste audit summary. (DOCX)

S4 File Data supplement. (DOCX)

S1 Data (CSV)

S2 Data (CSV)

## Abbreviations

CI: confidence interval
ICU: intensive care unit
OR: odds ratio
PICC: peripherally inserted central catheter
SOFA: sequential organ failure assessment
