# Supplementary material for: Patient harm associated with serial phlebotomy and blood waste in the intensive care unit: A retrospective cohort study
Source: PLoS One. 2021 Jan 13;16(1):e0243782. doi: 10.1371/journal.pone.0243782 (PMC7806151; doi:10.1371/journal.pone.0243782)
Supplement: S1 File — (DOCX) [file pone.0243782.s001.docx]

**S1 File. Manufacturer Tube Volumes**

**Table S1-1: Manufacturer vacuum tube volumes by tube colour.**

| Tube Colour | Common Lab Tests | Volume (mL) |
| --- | --- | --- |
| Red | Serum Drug Screen  EtOH Level  Therapeutic Drug Levels | 6 |
| Gold | General Biochemistry | 5 |
| Green | Ammonia  Lactate | 6 |
| Lavender | CBC  Blood Film  HbA1C | 4 |
| Blue | PT, aPTT  ROTEM | 4.5 |
| Pink | Blood Group  Antibody Testing | 6 |
| Syringe | Venous Blood Gas  Arterial Blood Gas | 3 |

*St. Michael’s Hospital, Toronto, Ontario, Canada - 2014/2015.*
