# Supplementary material for: Patient harm associated with serial phlebotomy and blood waste in the intensive care unit: A retrospective cohort study
Source: PLoS One. 2021 Jan 13;16(1):e0243782. doi: 10.1371/journal.pone.0243782 (PMC7806151; doi:10.1371/journal.pone.0243782)
Supplement: S2 File — (DOCX) [file pone.0243782.s002.docx]

**S2 File. Sensitivity Analysis**

**Sensitivity Analysis 1**

**Definition ICU Acquired Anemia / Nadir Hemoglobin value for Multivariable Logistic Regression:**

This analysis evaluates the sensitivity of the hemoglobin value used for the definition of ICU acquired anemia. Note patients with major bleeding events (Hb drop of 30 g/L within a 24 hours period) were excluded. Patients admitted with hemoglobin values below the cutoff were excluded.

**Table S2-1: Multivariable logistic regression for continuous and parametric predictors of ICU acquired anemia using different nadir hemoglobin values (Hb 90 g/L, 85 g/L, 80 g/L, 75 g/l).**

|  | Nadir Hb < 75 g/L  (N=348) | | Nadir Hb < 80 g/L  (N=327) | | Nadir Hb < 85 g/L  (N=308) | | Nadir Hb < 90 g/L  (N=289) | |
| --- | --- | --- | --- | --- | --- | --- | --- | --- |
|  | OR  (95% CI) | P | OR  (95% CI) | P | OR  (95% CI) | P | OR  (95% CI) | P |
| Daily phlebotomy volume, per mL | 1.03 (1.01 – 1.06) | <0.001 | 1.03 (1.01 – 1.06) | 0.002 | 1.03 (1.01 – 1.05) | 0.004 | 1.03 (1.01 – 1.05) | 0.008 |
| Age, yr | 1.01 (0.99 – 1.04) | 0.18 | 1.01 (0.99 – 1.04) | 0.19 | 1.01 (0.99 – 1.03) | 0.14 | 1.00 (0.98 – 1.02) | 0.83 |
| Sex (male) | 0.84 (0.42 – 1.66) | 0.62 | 0.71 (0.36 – 1.42) | 0.33 | 0.86 (0.45 – 1.65) | 0.65 | 0.61 (0.31 – 1.23) | 0.17 |
| ICU admission hemoglobin, g/L | 0.91 (0.88 – 0.93) | <0.001 | 0.91 (0.88 – 0.93) | <0.001 | 0.92 (0.89 – 0.94) | <0.001 | 0.90 (0.87 – 0.93) | <0.001 |
| ICU day 1 SOFA | 1.10 (0.98 – 1.24) | 0.09 | 1.17 (1.03 – 1.32) | 0.01 | 1.16 (1.03 -1.31) | 0.01 | 1.23 (1.08 – 1.40) | 0.002 |
| ICU length stay, d | 1.23 (1.15 – 1.31) | <0.001 | 1.21 (1.13 – 1.29) | <0.001 | 1.17 (1.1 – 1.24) | <0.001 | 1.16 (1.09 -1.23) | <0.001 |

CI = Confidence Interval, ICU = intensive care unit, SOFA = sequential organ failure assessment

**Sensitivity Analysis 2**

**Estimate of Blood Waste at Bedside:**

The below analysis uses daily phlebotomy volume received by the lab and excludes the estimate of blood wasted at the patient’s bedside during vascular access.

**Table S2-2: Multivariable logistic regression using daily phlebotomy volume received by the lab for testing (excluding blood wasted at bedside during vascular access) to predict 1) nadir hemoglobin < 80 g/L, 2) red cell transfusion in the ICU, and 3) hospital mortality. N=384 after exclusion of major bleeding events*.**

|  | 1) Nadir Hb < 80 g/L | | 2) Red Blood Cell Transfusion | | 3) Hospital Mortality | |
| --- | --- | --- | --- | --- | --- | --- |
|  | Odds Ratio Estimates  (95% CI)** | P | Odds Ratio Estimates  (95% CI)** | P | Odds Ratio Estimates  (95% CI)** | P |
| Daily phlebotomy volume**, per mL | 1.05 (1.02 – 1.09) | 0.001 | 1.05 (1.03 – 1.08) | <0.001 | 1.03 (1.01 – 1.05) | 0.005 |
| Age, yr | 1.02 (0.99 – 1.04) | 0.16 | 1.02 (1.00 – 1.04) | 0.04 | 1.03 (1.01 – 1.05) | 0.005 |
| Sex (male) | 0.70 (0.35 – 1.40) | 0.32 | 1.04 (0.59 – 1.86) | 0.89 | 1.05 (0.60 – 1.85) | 0.86 |
| ICU admission hemoglobin, g/L | 0.91 (0.88 - 0.93) | <0.001 | 0.93 (0.92 – 0.95) | <0.001 | 1.01 (1.00 – 1.20) | 0.12 |
| ICU admission SOFA score | 1.17 (1.04 – 1.32) | 0.01 | 1.15 (1.05 – 1.27) | 0.003 | 1.11 (1.01 – 1.21) | 0.03 |
| ICU length of stay, d | 1.21 (1.13 – 1.29) | <0.001 | 1.03 (1.01 – 1.06) | 0.002 | 1.02 (1.00 – 1.03) | 0.01 |

CI = Confidence Interval, ICU = intensive care unit, SOFA = sequential organ failure assessment

*Major Bleeding Definition: a drop in hemoglobin of > 30 g/L in a 24 hour period

**Excludes blood wasted at the bedside during vascular access (i.e. only blood received by lab for testing)
