# Supplementary material for: Patient harm associated with serial phlebotomy and blood waste in the intensive care unit: A retrospective cohort study
Source: PLoS One. 2021 Jan 13;16(1):e0243782. doi: 10.1371/journal.pone.0243782 (PMC7806151; doi:10.1371/journal.pone.0243782)
Supplement: S3 File — (DOCX) [file pone.0243782.s003.docx]

**S3 File. Waste Audit Summary**

A prospective blood waste audit was conducted at St. Michael’s Hospital in the medical-surgical ICU between February 26 and March 10, 2018. A total of 132 blood draws were observed over the course of four day-time shifts (9 AM to 5 PM), three day-evening shifts (2 PM to 10 PM), and three overnight shifts (11 PM to 7 AM). Draws per shift are summarized in [Figure S3-1](#Figure1). Average frequency of phlebotomy from each vascular access line and average blood draw volumes by line are summarized in [Table S3-1](#TableA2_1).


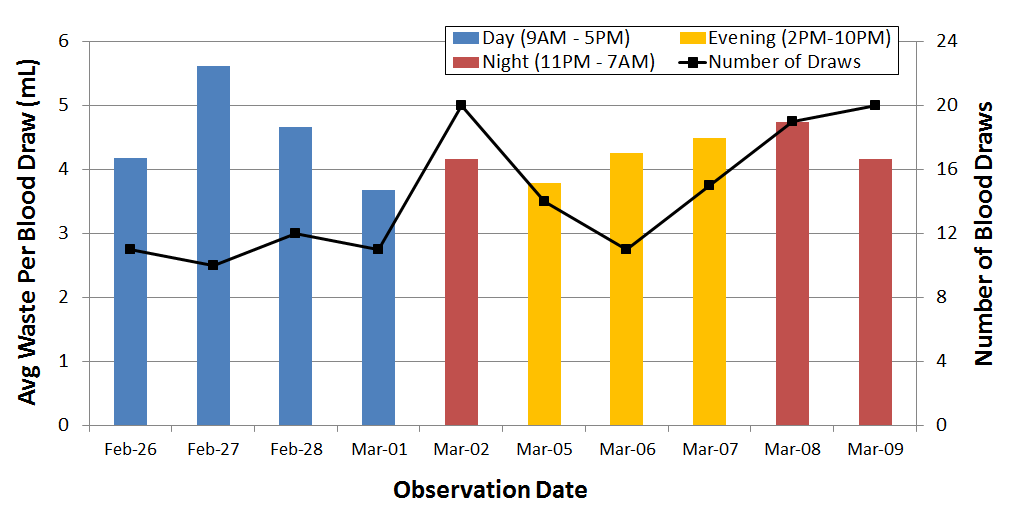


**Figure S3-1: Medical-surgical ICU prospective phlebotomy audit data**

**Table S3-1: Average blood waste per draw by line type.**

|  | **Number of Draws Audited (No., %)** | **Total Blood Waste Volume (mL)** | **Blood Waste per Draw (mL)** |
| --- | --- | --- | --- |
| Arterial Line | 109 (76%) | 424.0 | 3.9 |
| Central Venous Catheter (CVC) | 7 (5%) | 38.5 | 5.5 |
| Peripherally Inserted Central Catheter (PICC) | 16 (11%) | 100.0 | 6.3 |

**Equation for Estimated Blood Waste:**

Results summarized in [Figure S2-1](#Figure1) and [Table S2-1](#TableA2_1) were used to estimate bedside phlebotomy waste in the retrospective cohort (Sept 2014 to Aug 2015). Using the observed proportion of access to different vascular devices and average waste per device the following equation was generated:

**Equation 1**

| Blood Waste (mL)  =  (#draws)*(%draws Arterial Line)*(Avg. volume Arterial Line)  +  (#draws)*(%draws CVC)*(Avg. volume CVC)  +  (#draws)*(%draws PICC)*(Avg. volume PICC)  This equation is therefore:  Blood Waste (mL) = (#draws) * 76% * 3.9 + (#draws) * 5% * 5.5 + (#draws) * 11% * 6.3 |
| --- |
