# Supplementary material for: Patient harm associated with serial phlebotomy and blood waste in the intensive care unit: A retrospective cohort study
Source: PLoS One. 2021 Jan 13;16(1):e0243782. doi: 10.1371/journal.pone.0243782 (PMC7806151; doi:10.1371/journal.pone.0243782)
Supplement: S4 File — (DOCX) [file pone.0243782.s004.docx]

**S4 File. Data Supplement**

The following data elements are provided:

1. Logistic regression model data (with data dictionary)
   1. [Log_Reg_Data.csv]
2. Waste audit data
   1. [Waste_Audit_Data.csv]
3. Phlebotomy trend data (provided below)
4. Hemoglobin trend data (provided below)

**1. Logistic Regression Model Data**

| **Parameter** | **Description** | **Units** |
| --- | --- | --- |
| Maj_Bleed | Hb Drop > 30 g/L in 24 hrs | 1 = Yes, 0 = No |
| Sex | Male / Female | 1 = Female, 0 = Male |
| ICU_LOS | ICU length of stay | Days |
| HOSP_LOS | Hospital length of stay | Days |
| RBC_Tx | Any red cell transfusion in ICU | 1 = Yes, 0 = No |
| RBC_Tx_Num | Number of red cell transfusions in ICU | Number |
| Mortality | Hospital Mortality | 1 = Died, 0 = Lived |
| Nadir_Hb | Lowest hemoglobin in ICU | g/L |
| Day1_Hb | Admission hemoglobin | g/L |
| Nadir_Hb70 | Lowest Hb less than 70 | 1 = Yes, 0 = No |
| Nadir_Hb75 | Lowest Hb less than 75 | 1 = Yes, 0 = No |
| Nadir_Hb80 | Lowest Hb less than 80 | 1 = Yes, 0 = No |
| Nadir_Hb85 | Lowest Hb less than 85 | 1 = Yes, 0 = No |
| Nadir_Hb90 | Lowest Hb less than 90 | 1 = Yes, 0 = No |
| ICU_Phleb_Lab | Average ICU blood volume sent to lab for testing | mL/day in ICU |
| ICU_Phleb_Wst | Average ICU blood waste at bedside (estimate) | mL/day in ICU |
| ICU_Phleb_Tot | Average Total ICU blood volume (lab + waste) | mL/day in ICU |
| SOFA | Admission ICU Sequential Organ Failure Assessment Score | Number |
| SOFA_Mod | Admission ICU Sequential Organ Failure Assessment Score (Intubated GCS = 15/15) | Number |

*Note Age was removed prior to distribution in accordance with St. Michael’s Research Ethics Board requirements.

**Phlebotomy Trend Data:**

| ICU Day | Average Phleb  (mL) | Average  Waste  (mL) | %Waste | Average  Total  (mL) | Standard Dev | 95% CI |
| --- | --- | --- | --- | --- | --- | --- |
| 1 | 40.2 | 17.51 | 30% | 57.7 | 23.7 | 1.5 |
| 2 | 42.1 | 24.27 | 37% | 66.4 | 28.4 | 1.9 |
| 3 | 35.8 | 19.13 | 35% | 55 | 22 | 1.7 |
| 4 | 34 | 16.58 | 33% | 50.6 | 22.5 | 2 |
| 5 | 33 | 15.75 | 32% | 48.8 | 20.9 | 2.2 |
| 6 | 32.7 | 16.25 | 33% | 49 | 21.2 | 2.4 |
| 7 | 33.2 | 15.88 | 32% | 49.1 | 20.8 | 2.6 |
| 8 | 33.3 | 15.9 | 32% | 49.2 | 21.2 | 2.9 |
| 9 | 34.7 | 16.28 | 32% | 51 | 23.7 | 3.5 |
| 10 | 34.7 | 15.93 | 31% | 50.6 | 23.2 | 3.6 |
| 11 | 34.5 | 16.31 | 32% | 50.8 | 23.8 | 3.9 |
| 12 | 33.5 | 16.7 | 33% | 50.2 | 24.1 | 4.2 |
| 13 | 31.9 | 14.92 | 32% | 46.9 | 23 | 4.2 |
| 14 | 32.3 | 13.96 | 30% | 46.3 | 21.9 | 4.2 |
| 15 | 32.2 | 16.22 | 34% | 48.4 | 19.1 | 4 |
| 16 | 29.5 | 13.11 | 31% | 42.6 | 19 | 4.1 |
| 17 | 31.3 | 14.62 | 32% | 45.9 | 18.8 | 4.3 |
| 18 | 29.3 | 16.14 | 35% | 45.5 | 21.6 | 5.2 |
| 19 | 29.2 | 13.79 | 32% | 43 | 21 | 5.2 |
| 20 | 29 | 14.94 | 34% | 44 | 23.4 | 5.9 |
| 21 | 32.1 | 15.65 | 33% | 47.8 | 26.7 | 7.2 |
| 22 | 29.3 | 16.43 | 36% | 45.8 | 26 | 7.4 |
| 23 | 30.7 | 15.4 | 33% | 46.1 | 24.5 | 7.4 |
| 24 | 30.5 | 16.56 | 35% | 47 | 24.6 | 7.6 |
| 25 | 27.8 | 16.51 | 37% | 44.3 | 23 | 7.2 |
| 26 | 30.3 | 16.54 | 35% | 46.8 | 22.3 | 7.1 |
| 27 | 31.6 | 17.83 | 36% | 49.5 | 25.4 | 8.4 |
| 28 | 33.1 | 15.58 | 32% | 48.7 | 28.6 | 9.8 |
| 29 | 32.2 | 15.37 | 32% | 47.5 | 23.3 | 8.1 |
| 30 | 29 | 14.76 | 34% | 43.7 | 24 | 8.5 |
| 31 | 27.3 | 11.19 | 29% | 38.5 | 19.9 | 7.1 |
| 32 | 25.6 | 11.79 | 32% | 37.4 | 16.9 | 6.5 |
| 33 | 29.5 | 14.24 | 33% | 43.8 | 24.4 | 9.6 |
| 34 | 22 | 11.1 | 34% | 33 | 16 | 6.5 |
| 35 | 21.5 | 10.15 | 32% | 31.7 | 12.8 | 5.2 |
| 36 | 22.7 | 9.95 | 30% | 32.7 | 14.6 | 6.1 |
| 37 | 23.3 | 11.45 | 33% | 34.8 | 14.3 | 6.1 |
| 38 | 25.9 | 12.18 | 32% | 38.1 | 20.4 | 8.7 |
| 39 | 27.2 | 11.85 | 30% | 39 | 24.5 | 10.7 |
| 40 | 32.2 | 15.47 | 32% | 47.7 | 26 | 11.7 |
| 41 | 25.6 | 14.69 | 36% | 40.2 | 19 | 8.5 |
| 42 | 24.3 | 12.03 | 33% | 36.3 | 22.8 | 10.5 |
| 43 | 24.6 | 13.05 | 35% | 37.6 | 25.1 | 11.9 |
| 44 | 36 | 15.5 | 30% | 51.5 | 36.6 | 17.4 |
| 45 | 26.1 | 14.3 | 35% | 40.4 | 27.3 | 13.4 |
| 46 | 22.8 | 10.06 | 31% | 32.8 | 15.4 | 7.6 |
| 47 | 19.4 | 10.71 | 36% | 30.1 | 11.5 | 5.8 |
| 48 | 19.7 | 10.04 | 34% | 29.8 | 16.8 | 8.5 |
| 49 | 21.7 | 9.86 | 31% | 31.5 | 16.3 | 8.2 |
| 50 | 18.8 | 11.6 | 38% | 30.4 | 16.6 | 8.4 |

**Hemoglobin Trend Data**

| Admission Hb Strata | Hb > 120 g/L  (N=109) | Hb 120-100 g/L  (N=135) | Hb 100-80 g/L  (N=122) | Hb < 80 g/L  (N=62) |  |
| --- | --- | --- | --- | --- | --- |
| Day | Hb (g/L) | Hb (g/L) | Hb (g/L) | Hb (g/L) | COUNT |
| 1 | 135 | 111 | 91 | 72 | 426 |
| 2 | 126 | 105 | 88 | 76 | 405 |
| 3 | 119 | 100 | 85 | 76 | 391 |
| 4 | 116 | 98 | 85 | 77 | 371 |
| 5 | 114 | 97 | 86 | 78 | 315 |
| 6 | 113 | 96 | 85 | 77 | 255 |
| 7 | 111 | 95 | 83 | 76 | 204 |
| 8 | 109 | 93 | 83 | 76 | 171 |
| 9 | 105 | 91 | 82 | 78 | 149 |
| 10 | 105 | 90 | 81 | 79 | 134 |
| 11 | 102 | 87 | 81 | 76 | 122 |
| 12 | 101 | 85 | 78 | 77 | 111 |
| 13 | 95 | 84 | 79 | 76 | 97 |
| 14 | 99 | 87 | 80 | 79 | 88 |
| 15 | 102 | 81 | 76 | 79 | 74 |
| 16 | 99 | 81 | 77 | 78 | 64 |
| 17 | 93 | 84 | 77 | 79 | 60 |
| 18 | 96 | 85 | 80 | 80 | 50 |
| 19 | 90 | 85 | 80 | 83 | 48 |
| 20 | 98 | 83 | 81 | 81 | 48 |
| 21 | 92 | 84 | 78 | 79 | 41 |
| 22 | 85 | 83 | 80 | 78 | 33 |
| 23 | 83 | 84 | 78 | 79 | 37 |
| 24 | 89 | 80 | 79 | 74 | 31 |
| 25 | 86 | 83 | 81 | 76 | 27 |
| 26 | 87 | 81 | 81 | 79 | 27 |
| 27 | 83 | 80 | 82 | 75 | 29 |
| 28 | 86 | 78 | 81 | 83 | 29 |
| 29 | 88 | 77 | 76 | 84 | 24 |
| 30 | 84 | 79 | 79 | 81 | 27 |
| 31 | 88 | 78 | 76 | 82 | 26 |
| 32 | 81 | 76 | 83 | 75 | 21 |
| 33 | 86 | 76 | 80 | 79 | 21 |
| 34 | 86 | 78 | 77 | 82 | 19 |
| 35 | 82 | 77 | 84 | 83 | 18 |
| 36 | 86 | 76 | 75 | 81 | 18 |
| 37 | 87 | 76 | 77 | 82 | 15 |
| 38 | 81 | 75 | 77 | 82 | 18 |
| 39 | 91 | 73 | 77 | 79 | 17 |
| 40 | 77 | 77 | 70 | 76 | 14 |
| 41 | 92 | 78 | 72 | 80 | 15 |
| 42 | 77 | 77 | 75 | 81 | 11 |
| 43 | 84 | 79 | 80 | 82 | 12 |
| 44 | 91 | 75 | 77 | 82 | 12 |
| 45 | 85 | 74 | 80 | 81 | 12 |
| 46 | 88 | 76 | 77 | 75 | 10 |
| 47 | 91 | 74 | 77 | 76 | 11 |
| 48 | 80 | 82 | 76 | 76 | 11 |
| 49 | 88 | 85 | 67 | 71 | 9 |
| 50 | 111 | 85 | 76 | 79 | 10 |
